# The extracellular matrix controls stem cell specification and crypt morphology in the developing and adult mouse gut

**DOI:** 10.1242/bio.059544

**Published:** 2022-11-23

**Authors:** Rana Ramadan, Valérie M. Wouters, Sanne M. van Neerven, Nina E. de Groot, Tania Martins Garcia, Vanessa Muncan, Olivia D. Franklin, Michelle Battle, Karen Sue Carlson, Joshua Leach, Owen J. Sansom, Olivier Boulard, Mathias Chamaillard, Louis Vermeulen, Jan Paul Medema, David J. Huels

**Affiliations:** ^1^ Laboratory for Experimental Oncology and Radiobiology, Center for Experimental and Molecular Medicine, Cancer Center Amsterdam, University of Amsterdam, Meibergdreef 9, 1105 AZ, Amsterdam, The Netherlands; ^2^ Oncode Institute, Meibergdreef 9, 1105 AZ, Amsterdam, The Netherlands; ^3^ Department of Gastroenterology and Hepatology, Tytgat Institute for Intestinal and Liver Research, Amsterdam Gastroenterology Endocrinology and Metabolism, Amsterdam UMC University of Amsterdam, 1015 BK Amsterdam, The Netherlands; ^4^ The Medical College of Wisconsin, Department of Cell Biology, Neurobiology, and Anatomy, Milwaukee, WI 53226, USA; ^5^ The Blood Research Institute of Wisconsin, part of Versiti, and the Medical College of Wisconsin, Department of Internal Medicine, Milwaukee, WI 53226, USA; ^6^ Cancer Research UK Beatson Institute, Garscube Estate, Switchback Road, Glasgow, G61 1BD, UK; ^7^ Institute of Cancer Sciences, University of Glasgow, Garscube Estate, Switchback Road, Glasgow, G61 1QH, UK; ^8^ CNRS, Inserm, CHU Lille, Institut Pasteur de Lille, U1019 – UMR 8204 – Centre d'Infection et d'Immunité de Lille (CIIL), Université de Lille, 59019 Lille, France

**Keywords:** Crypt morphology, Extracellular matrix, Intestinal stem cells, Laminin

## Abstract

The rapid renewal of the epithelial gut lining is fuelled by stem cells that reside at the base of intestinal crypts. The signal transduction pathways and morphogens that regulate intestinal stem cell self-renewal and differentiation have been extensively characterised. In contrast, although extracellular matrix (ECM) components form an integral part of the intestinal stem cell niche, their direct influence on the cellular composition is less well understood. We set out to systematically compare the effect of two ECM classes, the interstitial matrix and the basement membrane, on the intestinal epithelium. We found that both collagen I and laminin-containing cultures allow growth of small intestinal epithelial cells with all cell types present in both cultures, albeit at different ratios. The collagen cultures contained a subset of cells enriched in fetal-like markers. In contrast, laminin increased Lgr5+ stem cells and Paneth cells, and induced crypt-like morphology changes. The transition from a collagen culture to a laminin culture resembled gut development *in vivo*. The dramatic ECM remodelling was accompanied by a local expression of the laminin receptor ITGA6 in the crypt-forming epithelium. Importantly, deletion of laminin in the adult mouse resulted in a marked reduction of adult intestinal stem cells. Overall, our data support the hypothesis that the formation of intestinal crypts is induced by an increased laminin concentration in the ECM.

## Teaser

**Summary:** ECM remodelling plays a role in the maintenance of intestinal stem cells and crypt morphology during development and in the adult mouse gut.

## INTRODUCTION

The small intestinal epithelium is a rapidly dividing tissue that renews itself from adult intestinal stem cells that reside at the bottom of intestinal crypts. The balance between proliferation and differentiation is finely controlled by growth factors and morphogenic signals, creating a niche at the bottom of the crypts that supports stem cell growth and suppresses differentiation (McCarthy et al., 2020a). The development of the *in vitro* organoid system brought mesenchymal growth factors into focus that were found to be essential for the growth of intestinal stem cells *in vitro*, and were then shown to have a similar role *in vivo*, e.g. R-spondin (Sato et al., 2009; Storm et al., 2016; Yan et al., 2017). Another requirement of the organoid system is an extracellular matrix (ECM)-derived hydrogel (Matrigel or basement membrane extracts), providing the required mechanical and signalling cues of the organoid surroundings. These matrices are derived from a mouse sarcoma, the Engelbreth–Holm–Swarm tumour, and are rich in laminin, collagen IV and entactin. The ECM can be divided into two main types: the interstitial matrix surrounding the tissue and the basement membrane, a thin ECM layer separating the epithelium from the interstitium. The role of the ECM goes beyond just providing a physical scaffold for tissues. Its dynamic remodelling is essential for organ development and in disease (Bonnans et al., 2014). For example, it has long been established that changes in the ECM have a fundamental role for the development of the mammary gland. Here, collagen I induces ductal branching and in turn causes a remodelling of the basement membrane, which is responsible for the differentiation of the mammary gland (Ghajar and Bissell, 2008; Keely et al., 1995). This ECM remodelling highlights different but coordinated roles for collagen I and the basement membrane in the development and formation of the mammary gland. This raises the possibility that, in the intestine, the interstitial matrix and the basement membrane fulfil similar roles in the development and maintenance of the stem cell niche. In fact, many efforts have been made to recapitulate events leading to crypt morphogenesis and the formation of stem cell compartments *in vivo* and *in vitro* (Sumigray et al., 2018; Kwon et al., 2020; Yang et al., 2021; Serra et al., 2019; Chang et al., 2008). Earlier studies already observed a fundamental remodelling of the intestinal ECM during gut development (Simo et al., 1991; Simon-Assmann et al., 1990). Spatial and temporal expression of the distinct laminin and integrin subunits has also been characterised in the human and murine intestines; however, its direct functional relevance on the epithelium is not well described (Simon-Assmann et al., 1994). In addition to laminin-rich hydrogel cultures (e.g. Matrigel), recent reports showed that intestinal epithelial cells can be maintained in a pure culture of fibrillar collagen type I, with the presence of stem cells as well as differentiated cells, suggesting that supplemented basement membrane components are not essential for the maintenance of intestinal stem cells (Jabaji et al., 2014; Wang et al., 2017). However, a recent study by Yui et al. (2018) showed that collagen type I induces a reprogramming of the adult intestine to a more fetal-like epithelium with a suppression of intestinal stem cell genes. This ECM remodelling becomes evident in colonic inflammation and regeneration where an increased expression and deposition of collagen type I and a reduction in laminin protein can be detected (Graham et al., 1988; Johnson et al., 2013; Schmehl et al., 2000). Furthermore, changes in ECM composition have been widely studied in relation to colorectal cancer onset, disease progression and metastasis (Winkler et al., 2020; Brauchle et al., 2018).

In this study, we compared in detail the influence of two different natural ECM-derived matrices with similar physical properties, the basement membrane (Matrigel, laminin or collagen IV) and the interstitial matrix (collagen I) on the intestinal epithelium. Both ECMs allowed growth of intestinal cells *in vitro* with all major cell types present in both cultures, although at different ratios. Collagen cultures induced a fetal-like expression program in a subset of cells and contained a reduced number of stem cells as well as Paneth cells. In contrast, laminin signalling (via ITGA6) was responsible for an increase in intestinal stem cells and induction of mor­phological crypt-like structures. These observations showed a striking similarity to the situation *in vivo*, where laminin deposition by the mesenchyme was enhanced during crypt formation, and deletion of laminin in adult mice resulted in stem cell loss and upregulation of fetal-like genes. These data support the idea that the deposition of laminin to the ECM by mesenchymal cells controls intestinal crypt development as well as stem cell maintenance *in vitro* and *in vivo*.

## RESULTS

### The ECM controls expression of fetal-like genes and stem cells

**Fig. 1.**
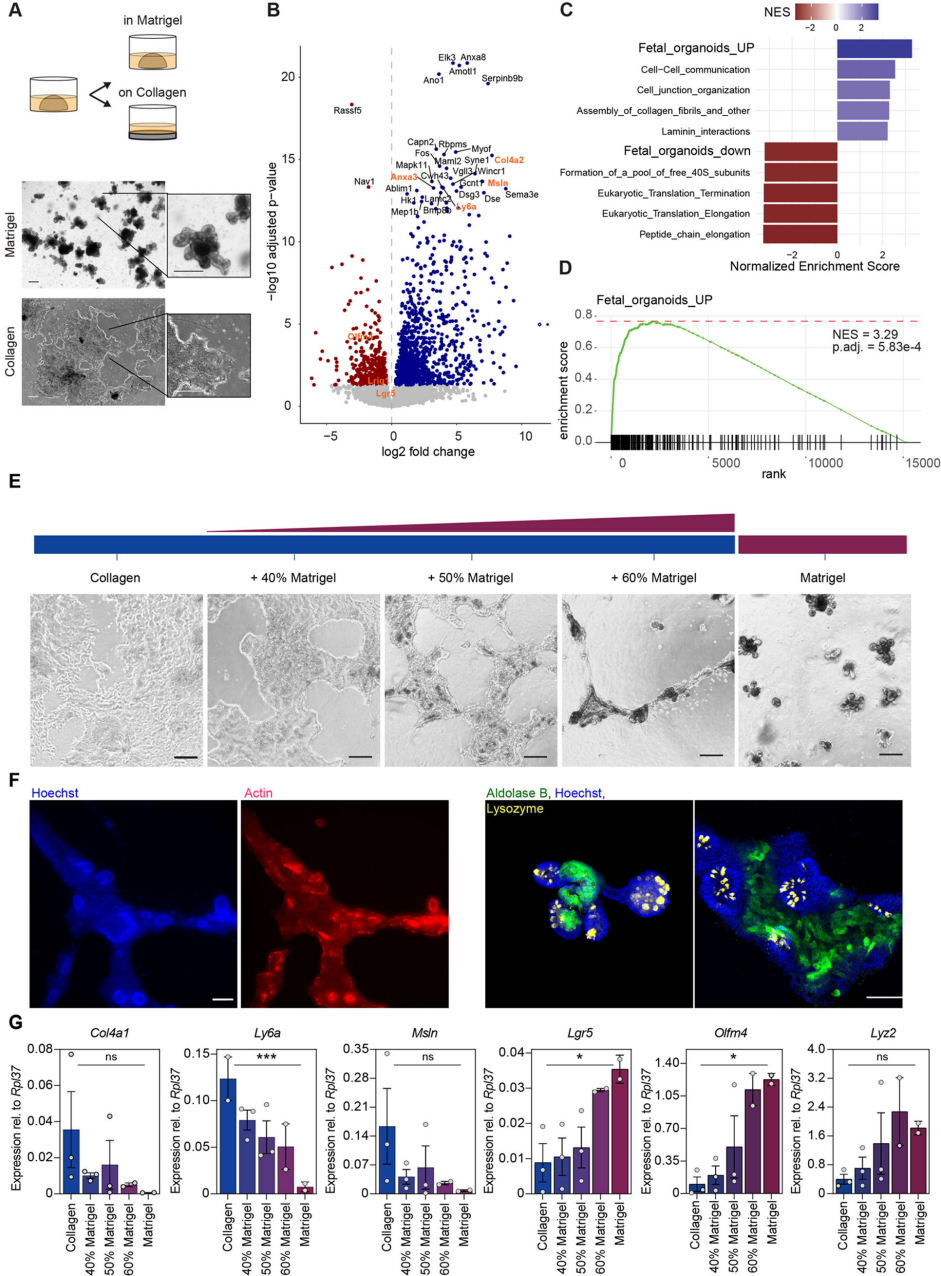
**ECM controls intestinal stem cell genes and fetal-like genes.** (A) Intestinal cells from adult wild-type mice were isolated and cultured for two passages in Matrigel. Matrigel-derived organoids were then seeded on a collagen layer for at least two passages before RNAseq (*n*=organoids from three different mice). Scale bar: 200 µm. (B) Volcano plot shows significantly (*P*_adj_<0.05) upregulated (blue) and downregulated (red) genes in collagen versus Matrigel cultures. The 30 most significant deregulated genes and intestinal stem cell genes (*Lgr5*, *Olfm4* and *Lrig1*) are labelled. Fetal-like genes and intestinal stem cell genes are highlighted in orange. (C) Gene set enrichment analysis was performed with reactome pathway signatures and fetal-organoid signature (Yui et al., 2018). The top five upregulated and downregulated pathways [ordered by normalized enrichment score (NES)] are shown. (D) Fetal signature is highly enriched in collagen cultures. (E) Collagen cultures were plated on different matrices: collagen with or without the addition of Matrigel, or pure Matrigel. Scale bar: 100 µm. (F) Immunofluorescence (left panels) of collagen+50% Matrigel shows crypt-like protrusions with apical accumulation of actin. Immunofluorescence (right panels) of collagen+50% Matrigel (left) and Matrigel (right) show crypt-like protrusions containing lysozyme+ Paneth cells and surrounding aldolase B+ enterocytes. Scale bars: 100 µm. (G) qRT-PCR analyses show decrease of fetal-like genes (*Col4a1*, *Ly6a*, *Msln*) and upregulation of intestinal stem cell genes (*Lgr5*, *Olfm4*) and Paneth cell genes (*Lyz2*). Each dot represents an independent experiment. Data show the mean±s.e.m. Simple linear regression. ns, not significant; *P<0.05; ***P<0.001.

To study the impact of the ECM on the small intestinal epithelium, we plated organoids in Matrigel and collagen type I. Matrigel is a basement membrane extract that mainly consists of laminin, collagen IV and entactin. Collagen type I is the main component of the interstitium and is the most abundant protein in mammals. We purified small intestinal crypts from adult wild-type mice and grew them as organoids as previously described (Sato et al., 2009). After establishment, we plated organoids on a pure layer of collagen I or in Matrigel (Jabaji et al., 2014; Wang et al., 2017) (Fig. 1A). As the three-dimensional (3D) growth of intestinal cells embedded in collagen is only possible with addition of Wnt3a, we focused on the two-dimensional (2D) model, in which cells are plated on a thick layer of collagen I and can be maintained in the same medium as Matrigel cultures (Yui et al., 2018). Recently, synthetic matrices have been described that allow stem cell survival and proliferation or differentiation by changing the stiffness of the hydrogels, indicating that physical properties influence the epithelial cell composition (Gjorevski et al., 2016). To avoid stiffness-related effects, we chose the collagen type I concentration that showed a similar stiffness as that of Matrigel (Fig. S1A). Similar to the Matrigel cultures, collagen cultures required R-spondin for long-term culture (Fig. S1B). We performed RNA sequencing (RNAseq) to compare the effect of the distinct ECMs on the epithelial cells and detected marked differences in the gene expression profiles (Fig. 1B). Several of the most significantly upregulated genes in the collagen cultures were those characteristic of fetal organoids (e.g. *Anxa3*, *Ly6a/Sca1*, *Msln* and *Col4a2*) (Mustata et al., 2013). In contrast, genes expressed in adult intestinal stem cells were downregulated (e.g. *Olfm4* and *Lgr5*). To analyse the differentially expressed genes in an unbiased manner, we performed gene set enrichment analysis. This confirmed a striking upregulation of fetal organoid-associated signatures in the collagen cultures and a significant downregulation of adult organoid genes (Fig. 1C,D), confirming earlier results in a 3D collagen system with the addition of Wnt3a (Yui et al., 2018).

Importantly, the effect of ECM components on gene expression was not due to difference in morphology (2D collagen versus 3D Matrigel) but truly defined by the type of ECM, as cells grown in a 3D collagen environment were transcriptionally similar to the 2D collagen cultures (Fig. S1C-E). Specifically, the fetal signature was highly upregulated in both collagen cultures (2D and 3D) and adult genes were downregulated. Interestingly, addition of Wnt3a did not influence the expression of fetal genes but downregulated several genes of the adult organoid signature, which were mostly associated with Paneth cells (Fig. S1D). Principal component analysis of the global transcriptome showed that the collagen I cultures cluster together, independent of whether they were grown in 2D or 3D (Fig. S1E).

To further investigate the direct influence of ECM composition on the intestinal stem cells and expression of fetal-like genes, we plated collagen-grown cells on an ECM with increasing concentrations of Matrigel (Fig. 1E). Cells plated on collagen alone always grew as a flat 2D layer. We then mixed increasing concentrations of Matrigel into the collagen mixture, while maintaining the concentration of collagen I in the matrix. Strikingly, we observed crypt-like protrusions emerging from the epithelial network when 50% or more Matrigel was added to the collagen, whereas lower concentrations of Matrigel did not result in morphological changes (Fig. 1E). To verify whether the protrusions in the 50% Matrigel condition were indeed crypt-like structures, we performed actin staining, which marks the apical side of crypts, and staining for Paneth cells (O'Rourke et al., 2016). These protrusions were enriched for actin on the apical membrane and contained lysozyme-positive Paneth cells (Fig. 1F), highlighting a crypt-like morphology and compartmentalisation. Moreover, we performed staining for aldolase B, a component of the glycolytic pathway known to be upregulated in the villus compartment, to localise differentiated cells in our 50% Matrigel cultures (Chang et al., 2008; Lukonin et al., 2020). The flat epithelium surrounding the crypt-like structures accommodated aldolase B-positive enterocytes (Fig. 1F). In line with the morphological changes, we observed that increasing concentrations of Matrigel reduced the expression of fetal-like genes and increased Paneth cell and stem cell gene expression in a dose-dependent manner (Fig. 1G), despite the presence of the same collagen I concentration. Interestingly, we did not observe a similar change in absorptive enterocyte or goblet cell gene expression (*Alpi* and *Muc2*, respectively), nor an overall change in Wnt pathway activation (*Axin2*) when changing the ECM composition (Fig. S1F). This suggests that the increased basement membrane components have a dominant effect over collagen, resulting in induction of a crypt-like morphology and increased stem and Paneth cells.

### ECM components impact intestinal cell populations

**Fig. 2.**
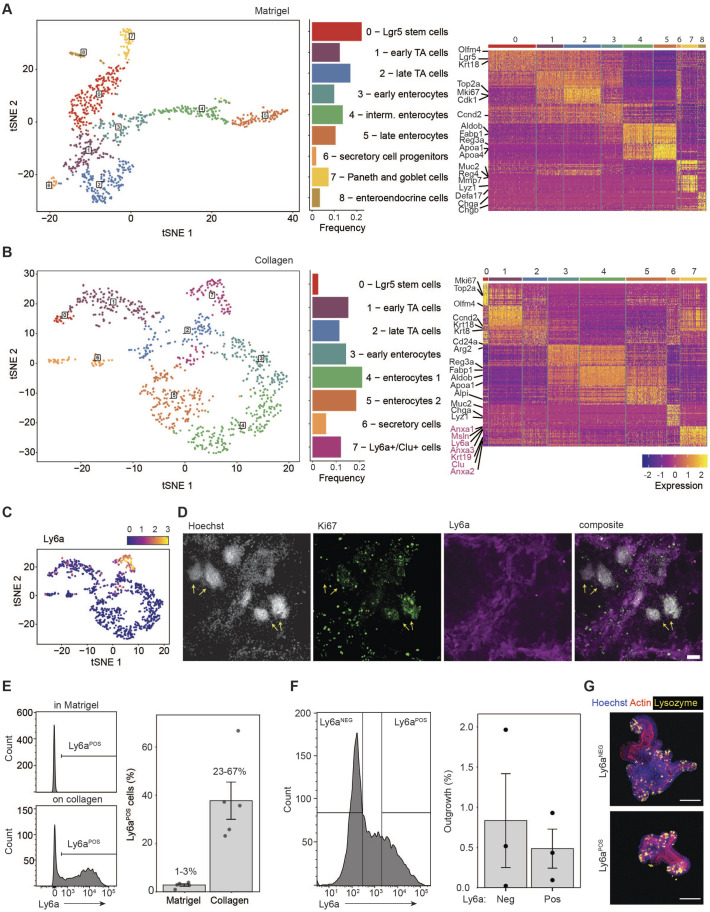
**Distinct fetal-like cell population in collagen cultures with stem cell capacity.** (A) Clustering of Matrigel-derived organoids at day 4 after passaging. The frequency plot shows the proportion of cells assigned to each cluster. The heatmap shows the top 30 differentially expressed genes in each cluster and labels of the characteristic genes of intestinal cell types. (B) Clustering of collagen-derived cells at day 4 after passaging. The frequency plot shows the proportion of cells assigned to each cluster. The heatmap shows the top 30 differentially expressed genes in each cluster and labels of the characteristic genes of intestinal cell types. Fetal-like genes are labelled purple. Cells for Matrigel and collagen single-cell RNAseq were derived from the same organoid line. (C) Ly6a expression in t-distributed stochastic neighbor embedding (tSNE) of collagen cultures. (D) Immunofluorescence of collagen culture, stained for nuclei (Hoechst), proliferation (Ki67) and a protein expressed by a fetal-like gene (Ly6a). Yellow arrows indicate the densely populated regions. Scale bar: 100 µm. (E) Flow cytometry of Ly6a-APC in Matrigel and collagen cultures. Each dot represents an independent experiment Data show the mean±s.e.m. *n*=5. (F) Ly6a-negative and -positive cells from collagen cultures were sorted and plated in Matrigel. Organoids were counted after 1 week; each dot represents an independent experiment. Data show the mean±s.e.m. *n*=3. (G) Immunofluorescence of organoids derived from Ly6a-sorted cells, stained for nuclei (Hoechst, blue), actin (red) and Paneth cells (lysozyme, yellow). Scale bar: 100 µm.

To gain further insight into the different cell populations, we performed single-cell RNAseq of collagen and Matrigel cultures. The cells present in Matrigel cultures could be clustered into nine distinct groups that could be related to known cell lineages of the small intestine. The heatmap (Fig. 2A) shows the most differentially expressed genes in each cluster, and characteristic genes that were used to assign the most probable cell type to each cluster are highlighted. A large proportion of cells were characterised by the expression of *Lgr5* as well as proliferation genes (*Lgr5-*expressing stem cell and transit-amplifying (TA) cell clusters). Three clusters belong to absorptive enterocytes, probably marking the different stages of development, as has been previously observed *in vivo* (Haber et al., 2017) (Fig. S2A – *Apoa1*). Even rare enteroendocrine cells were identified as a distinct cell cluster in our data (Fig. S2A – *Chga*). The heatmap also highlights several clusters belonging to proliferative progenitors that are in the process of differentiation, e.g. early absorptive enterocytes and secretory cell progenitors (Fig. 2A).

Intriguingly, in the collagen cultures, eight different cell clusters were distinguished, which represented most of the cell lineages that are present in Matrigel cultures (Fig. 2B). However, we observed fewer stem cells, TA cells and cells of secretory cell lineages (Fig. S2B). Due to their reduced number in this sample, enteroendocrine cells and Paneth/goblet cells formed a single cluster but were reliably de­tected by the expression of the characteristic genes in these cell types (Fig. S2B – *Chga* and *Lyz1/Muc2*). The distinct composition was further confirmed by immunofluorescence, which also revealed a distinct spatial distribution of Paneth cells and enterocytes in the collagen cultures (Fig. S3A).

Strikingly, one unique cluster was found on collagen that is not present in the Matrigel cultures. This cluster was characterised by the fetal-like genes *Ly6a/Sca1*, *Clu*, *Msln*, *Anxa1* and *Anxa3* (Fig. 2B,C; Fig. S3B). Importantly, expression of these genes was restricted to this cluster and was not detected in the other cell types (Fig. S3B). Interestingly, analysis of the combined cells from collagen and Matrigel cultures showed that all the secretory cells, e.g. Paneth and goblet cells, clustered together irrespective of their cell type, independent of whether they originated from collagen or Matrigel cultures. This suggests a similar transcriptional and differentiation profile in these cells that is unaffected by the ECM composition (Fig. S3C). We next focused on the fetal-like cell cluster found only in the collagen cultures. Staining for Ly6a showed that these cells are located around the densely packed cell regions containing Paneth cells (Fig. 2D; Fig. S3A), indicating a spatially distinct cell type. Notably, Ly6a+ cells as well as the densely populated cell regions stained positive for the proliferation marker Ki67 (Fig. 2D), suggesting that Ly6a− and Ly6a+ cells both sustain proliferation of the cultures. Analysis of the collagen and Matrigel cultures via flow cytometry confirmed that collagen cultures contained a reduced number of Lgr5-GFP cells and a large fraction of Ly6a+ cells (Fig. S3D). Overall, we observed Ly6a+ cells in varying numbers in the collagen cultures, but consistently at negligible low percentages in the Matrigel cultures (Fig. 2E). Furthermore, we did not observe any overlap of Lgr5-GFP and Ly6a staining, confirming that the Ly6a+ cells were a truly distinct cell population (Fig. S3E). Ly6a/Sca1+ as well as Clu+ cells have recently been described to be involved in the repair of the intestinal epithelium after damage and irradiation (Ayyaz et al., 2019; Yui et al., 2018). To test whether this *in vitro*-derived cell population has a similar stem cell potential, we sorted Ly6a-positive and -negative cells from collagen cultures and embedded them in Matrigel. Both cell populations had a similar capacity to form organoids (Fig. 2F), could be passaged and were morphologically indistinguishable from each other, as was previously demonstrated by Yui et al. (2018). Importantly, the Ly6a+ cell-derived organoids also contained Paneth cells and showed that the Ly6a+ cells have true stem cell capabilities (Fig. 2G).

### Laminin controls intestinal stem cell fate

**Fig. 3.**
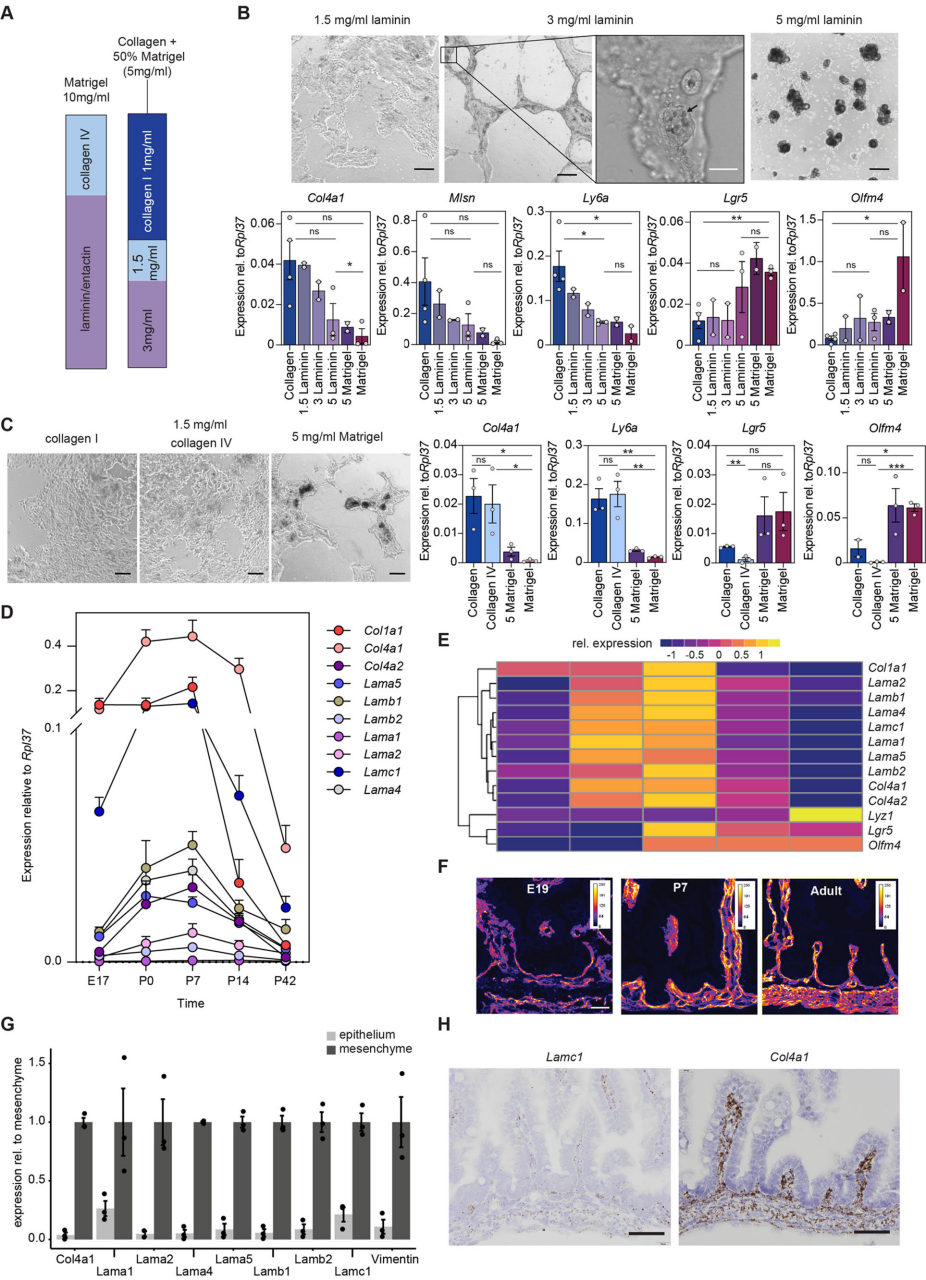
**Laminin induces crypt-like structures and is upregulated during crypt formation *in vivo*.** (A) Matrigel mainly consists of laminin and collagen IV. Single basement membrane components (laminin/entactin or collagen IV) were added to a 1 mg/ml collagen hydrogel. (B) Cells grown on collagen were plated on collagen I with laminin (1.5 mg/ml, 3 mg/ml, 5 mg/ml) or with Matrigel (5 mg/ml), or collagen or Matrigel alone. Laminin induced morphological changes, as well as a similar downregulation of fetal-like genes (*Col4a1*, *Ly6a*, *Msln*) and upregulation of stem cell markers (*Lgr5*, *Olfm4*). Black scale bar: 200 µm. White scale bar: 50 µm. Black arrow indicates Paneth cells in a crypt-like protrusion. (C) Cells grown on collagen were plated on collagen I, collagen I with 1.5 mg/ml collagen IV, collagen I with 5 mg/ml Matrigel, or Matrigel alone, and analyzed for gene expression by qRT-PCR. Scale bar: 100 µm. (D) qRT-PCR analysis of intestinal tissue at different timepoints during development. *n*=3 mice per time point for *Col4a2* and *n*=5 mice for all other genes. Data show the mean±s.e.m. (E) Heatmap of qRT-PCR averages scaled for each gene showing that most basement membrane genes cluster together, separate from collagen I (*Col1a1*) and laminin β1 (*Lamb1*) and are specifically upregulated at P0 (birth) and P7. Intestinal stem cell genes (*Lgr5*, *Olfm4*) are upregulated at P7. Paneth cell genes (*Lyz1*) appear later at P14 and increase further until P42. (F) Pan-laminin immunofluorescence shows increased laminin levels during crypt development (E19, P7 and adult). *n*=3 mice. Scale bar: 45 µm. (G) Intestinal crypts (without villi) at P7 were EDTA-separated for epithelium and mesenchyme. *Vimentin* shows enrichment of mesenchymal cells that are absent in the epithelial fraction. Laminin and collagen IV genes are highly expressed in the mesenchyme with little contribution of epithelial cells. (H) RNA *in situ* hybridisation (RNAscope) for *Lamc1* and *Col4a1* at P0 shows predominant mesenchymal expression. Scale bars: 50 µm. For all bar graphs (B,C,G), each dot represents an independent experiment; data show the mean±s.e.m. Unpaired two-tailed *t*-test; ns, not significant; *P<0.05; **P<0.005; ***P<0.001.

Having estab­lished that Matrigel and collagen affect the number of intestinal stem cells and fetal-like cells in organoid cultures, we asked which component of Matrigel is responsible for the observed effect. Matrigel is a basement membrane extract and consists mainly of laminin and collagen IV. As the most striking morphological effect was observed when 50% Matrigel was added to the collagen hydrogel, we tried to reproduce this effect by providing either laminin or collagen IV separately (Fig. 3A). Laminin was able to induce the same morphological as well transcriptional response as Matrigel in a dose-dependent manner (Fig. 3B). In contrast, the addition of collagen IV did not result in any morphological or transcriptomic changes (Fig. 3C). Furthermore, cells embedded in pure laminin hydrogels grew as 3D organoids, with similar characteristics as those grown in Matrigel (Fig. S1G). These data indicate that laminin is the main component responsible for the induction of the crypt-like structures and expression of genes associated with adult intestinal stem cells.

We noticed parallels between the developing small intestine *in vivo* and our *in vitro* system. Before birth, the mouse small intestine only contains villi and proliferating intervillus structures. Cells from both these compartments can contribute to the pool of adult stem cells after birth (Guiu et al., 2019). Around 2 weeks after birth, the final crypt structures have formed and mature Paneth cells can be detected (Bry et al., 1994; Calvert and Pothier, 1990; Dehmer et al., 2011). Therefore, we wondered whether the development of intestinal crypts *in vivo* showed a similar change in the ECM. We analysed the small intestine of wild-type mice from embryonic (E) day 17 until 6 weeks after birth [postnatal (P) day 42]. Intriguingly, a dramatic change around birth in the expression of most laminin isoforms (*Lama*, *Lamb* and *Lamc* genes) as well as collagen IV (*Col4a1*, *Col4a2*) was observed (Fig. 3D). This spike in expression could be detected for 1 week, after which the levels of expression reduced. Collagen type I (*Col1a1*), *Lamb2* and *Lama1* did not follow the same expression burst at P0 as observed with the other basement membrane components (Fig. 3D,E). Immunofluorescence staining for laminin further validated an increase in protein levels from E19 to P7 (Fig. 3F). Next, we aimed to determine which cells were responsible for the increased expression of the basement membrane components. We separated the epithelium from the mesenchyme of wild-type mice at 1 week after birth (P7). All basement membrane components were predominantly expressed in the mesenchyme and only low expression was found in the epithelium (Fig. 3G). RNA *in situ* hybridisation for laminin γ1 (*Lamc1*) and collagen IV (*Col4a1*) confirmed a specific upregulation exclusively in the mesenchyme (Fig. 3H; Fig. S4A). To visualise the laminin network, we cleared and stained the small intestine of a mouse 1 week after birth (P7). Laminin was detected surrounding the nascent crypts as well as villi (Fig. S4B). This indicates that the dramatic re­modelling of the ECM during crypt formation is driven by the mesenchyme and not the epithelium.

### Laminin signalling controls intestinal epithelium change via ITGA6

**Fig. 4.**
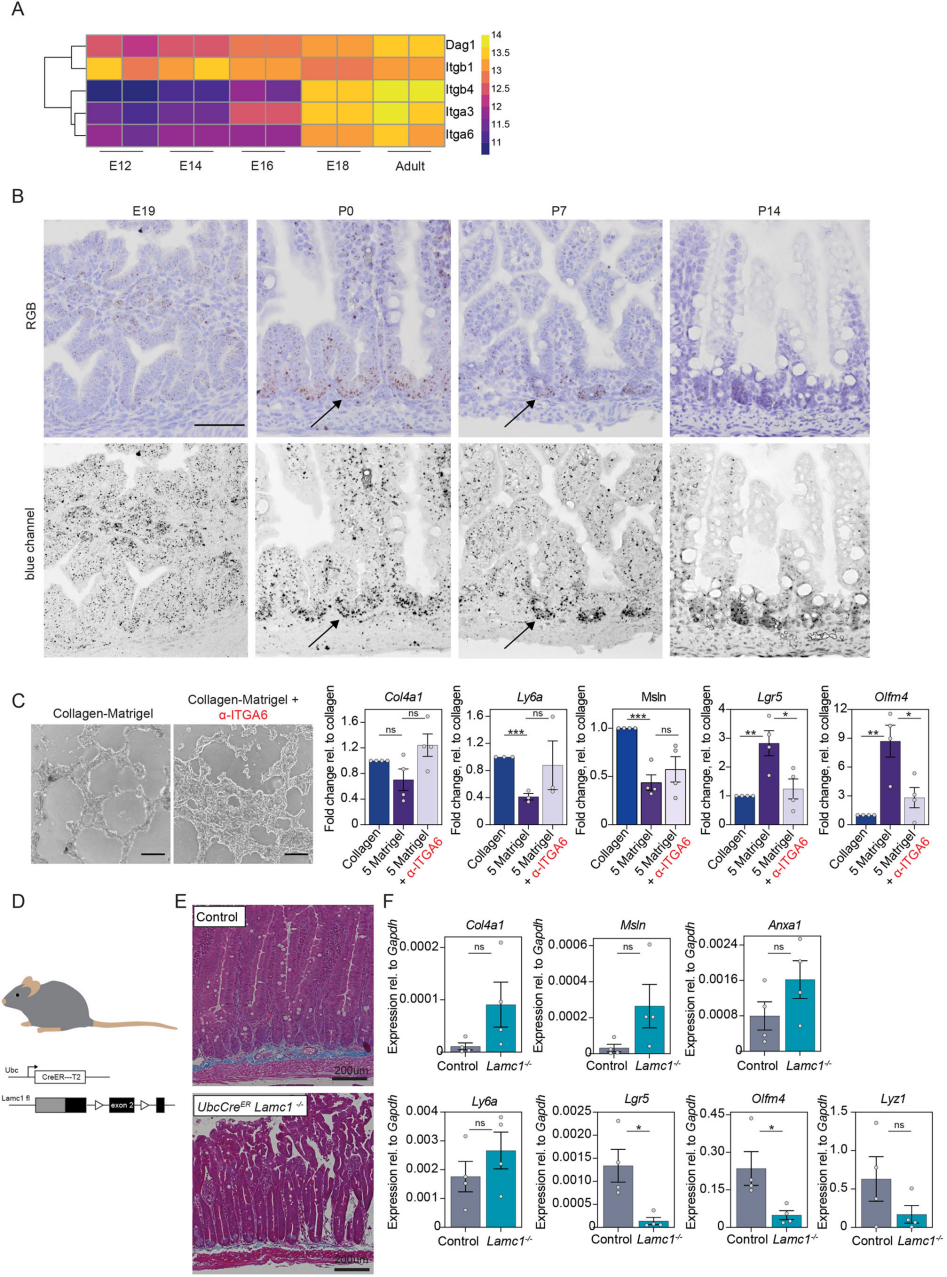
**Laminin signalling, via integrin α6, induces crypt formation *in vitro* and controls intestinal stem cells *in vivo*.** (A) An RNAseq dataset (GSE115541, Kumar et al., 2019) consisting of epithelial intestinal cells during development shows specific upregulation of the laminin receptor-encoding genes *Itga6*, *Itga3*, *Itgb4* and *Dag1* from E18 onwards. (B) RNA *in situ* hybridisation for *Itga6* shows increased expression at P0, with accumulation at the intervillus region. The bottom row shows blue channel images for increased contrast of the RNA *in situ* signal. Black arrows indicate inter-villus regions and nascent crypts. Scale bar: 50 µm. (C) Cells were grown on collagen and then seeded on collagen I or collagen I with 5 mg/ml Matrigel (5 Matrigel) mixture with or without addition of the ITGA6 neutralising antibody (α-ITGA6). Inhibition of ITGA6 blocks downregulation of fetal-like markers and blocks increase of stem cell markers. Each dot represents an independent experiment. *n*=4. All values are relative (fold change) to their collagen control. Scale bar: 1000 µm. (D) *Ubc-CreERT2 Lamc1loxP/loxP* adult mice (>6 weeks old) were injected with tamoxifen to delete *Lamc1* throughout the body, and mice were analysed 21 days after injection. (E) Trichrome staining shows an epithelial phenotype after deletion of *Lamc1*. Scale bar: 200 µm. (F) Stem cell markers were downregulated after deletion of *Lamc1*. *n*=4 mice per group. Each dot represents independent experiment. Data show the mean±s.e.m. Unpaired two-tailed *t*-test; ns, not significant; *P<0.05; **P<0.005; ***P<0.001.

Our data revealed a potent induction of the adult stem cell program and morphological changes by the addition of laminin, and that formation of intestinal crypts in gut development coincides with a temporal increase in laminin production by the mesenchyme. To gain further insight into the molecular signalling between the epithelium and the basement membrane components, we evaluated the expression of their receptors during development. The key family of receptors for the ECM are integrins and dystroglycan receptors. We analysed a publicly available dataset of intestinal epithelial cells during development for expression changes in integrins and dystroglycan-encoding genes (Kumar et al., 2019). Strikingly, at E18, a specific upregulation of *Itga6*, *Itgb4*, *Dag1* and *Itga3* was observed (Fig. 4A), whereas all other integrins were only lowly expressed and did not change during development (Fig. S4C). ITGA6 (together with ITGB4) is a known receptor for laminin (Lee et al., 1992). RNA *in situ* hybridisation for *Itga6* confirmed the previous analysis and showed a marked upregulation of *Itga6* after birth (Fig. 4B). Remarkably, the expression was even more pronounced in the intervillus region and stayed enriched in the crypts compared to villi. Next, we asked whether ITGA6 was mechanistically involved in the laminin-induced phenotypic change. Blocking ITGA6, in our previously described *in vitro* assay, diminished the induced morphological changes and prevented the upregulation of intestinal stem cell genes (Fig. 4C). Given this central role of laminin signalling during development, we evaluated the importance of laminin-epithelial signalling in the adult intestine *in vivo*. The laminin γ-chain is common to all laminin heterotrimeric proteins and was the highest expressed laminin gene during development (Fig. 3D). In a previous study, ubiquitous knockout of *Lamc1* in mice mainly affected the intestine and was lethal to adult mice after 24 days from induction. Drastic morphological changes were observed in mutant murine intestine, such as the epithelium separating from the mesenchyme. Importantly, *Lamc1* knockout did not result in the upregulation of alternative laminin γ subunits (Fields et al., 2019). We crossed the *Lamc1* flox mouse to a ubiquitously expressing Cre mouse to delete laminin expression in adult mice as previously reported (Fig. 4D) (Fields et al., 2019). Based on our *in vitro* model, we predicted that the depletion of laminin would result in a decrease of stem cells and Paneth cells, as well as an upregulation of fetal-like genes. Indeed, 3 weeks after the deletion of *Lamc1*, the intestinal epithelium showed a marked downregulation of the stem cell markers *Lgr5* and *Olfm4* and an upregulation of fetal-like genes in line with our *in vitro* experiments (Fig. 4E,F). *Lamc1* recombination was also performed *in vitro* in organoids cultured in Matrigel domes. No phenotypic or genotypic changes were observed in laminin γ1*-*deficient organoids when laminin was supplemented by an external source, confirming that the epithelial source of laminin γ1 was dispensable for crypt and stem cell maintenance (Fig. S4D).

**Fig. 5.**
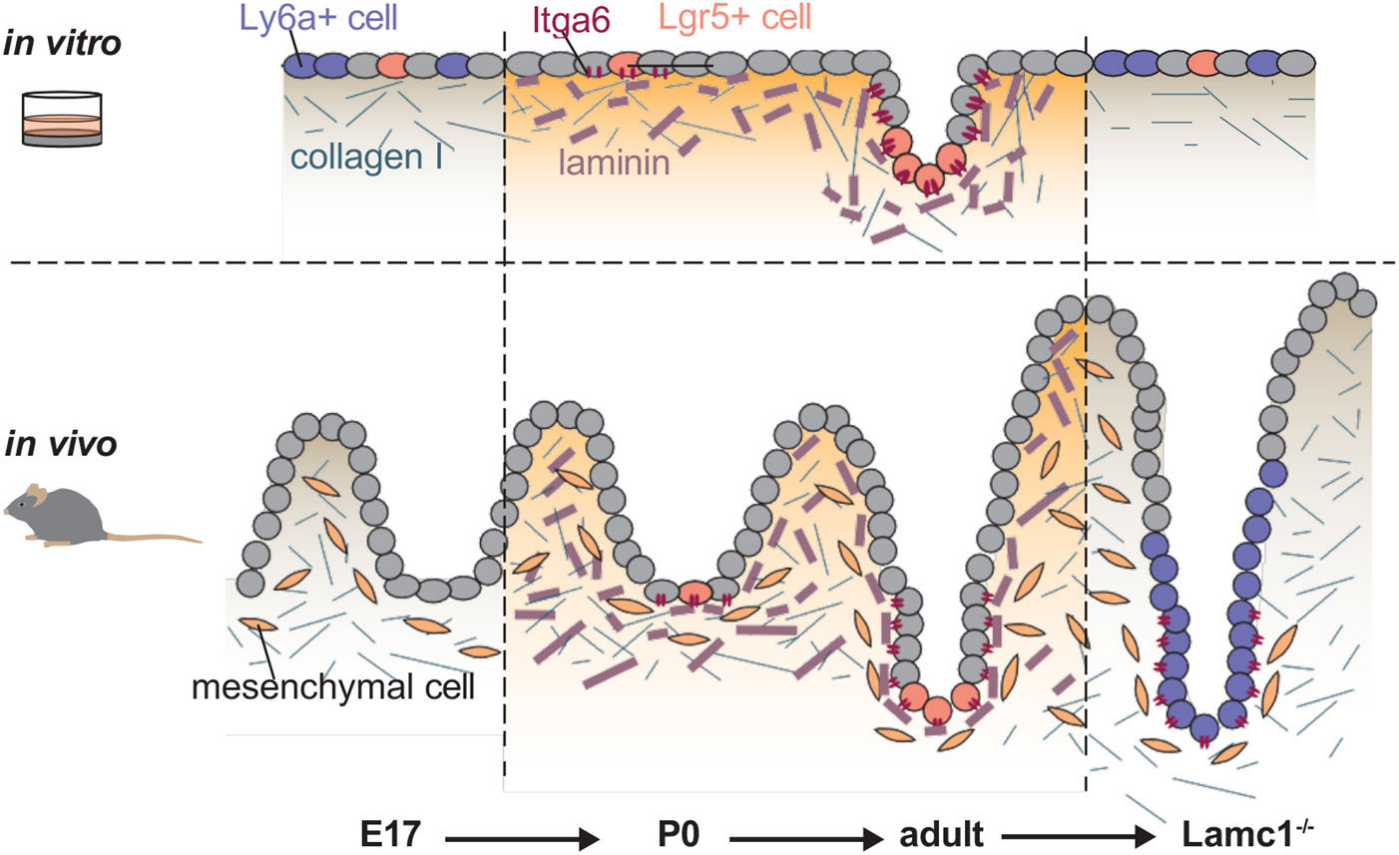
**Graphical abstract.** Parallels between *in vitro* cultures with different ECMs and *in vivo* crypt formation and homeostasis. *In vitro*, adult intestinal cells contain fetal-like cells (Ly6a+) and few intestinal stem cells (Lgr5+) and grow exclusively in a 2D layer. Addition of laminin induces an increase of Lgr5+ cells and Paneth cells (via ITGA6), resulting in a morphological crypt formation. *In vivo*, the mesenchyme dramatically remodels the ECM and increases laminin concentration. Concurringly, epithelial cells in the inter-villus region upregulate expression of the laminin receptor ITGA6 and progress to form intestinal crypts. Even in the adult, intestinal crypts rely on laminin signalling, as depletion of laminin results in a fetal-like epithelium.

Overall, these data support the hypothesis that the ECM composition, as orchestrated by the mesenchyme, induces and maintains crypt formation as well as intestinal stem cell specification in the developing and adult intestine (Fig. 5).

## DISCUSSION

How ECM remodelling influences intestinal tissue morphology during development and controls lineage specification is a topic of debate. In recent years, the focus on the intestinal niche has shifted considerably. With the development of organoid technology, the main focus has been on Paneth cells as the key part in establishing an intestinal stem cell niche (Sato et al., 2011, 2009). Careful analysis *in vivo*, however, showed that Paneth cells are dispensable and indicated that mesenchymal cells were the main provider of the intestinal niche (Durand et al., 2012; Kabiri et al., 2014; Kim et al., 2012). Furthermore, even organoid cultures relied on the addition of growth factors that are usually secreted by the mesenchyme (e.g. R-spondin). More recently, the focus shifted onto this mesenchymal niche and the different subtypes of fibroblasts that constitute this niche *in vivo* (McCarthy et al., 2020a). Next to secreted growth factors, such as Wnt ligands and R-spondin, data support a strong role for these mesenchymal cells in controlling the composition of the extracellular matrix *in vivo* and, there­fore, points to a potential role for the creation of a niche (Ormestad et al., 2006; Simo et al., 1991; Simon-Assmann et al., 1994, 1990). Here, we show that both the interstitial matrix (collagen I) as well as the basement membrane matrix (Matrigel/laminin) support the long-term growth of the intestinal epithelium with the presence of all major cell lineages. However, morphological crypt-like structures were only observed when the ECM contained a high concentration of laminin (and not collagen IV), supporting previous findings in other *in vitro* hydrogel settings (Broguiere et al., 2018). This is despite the presence of Lgr5+ stem cells and Paneth cells in the collagen cultures, which are shown to be responsible for crypt budding in Matrigel grown organoids (Serra et al., 2019). An interesting question that arises is whether the presence of collagen I or the lack of laminin signalling induces the fetal-like reprogramming. In a recent study from the Liberali lab, the authors showed that organoid growth in Matrigel from single cells undergoes a similar, transient fetal-like cell state, which then declines as Lgr5+ intestinal stem cells and Paneth cells appear (Serra et al., 2019). This could indicate that cells taken out of their ECM environment need some time to adapt to the laminin-rich environment and hence undergo a temporary fetal-like state. Therefore, a transient disruption of laminin signalling might already induce the fetal-like response. Our data also indicates that increasing concentrations of laminin revert the fetal-like state in the collagen cultures, despite an unchanged collagen concentration.

Overall, our data support the hypothesis that the basement membrane produced by the epithelium is not sufficient to increase stem cell numbers and induce a morphological crypt formation and, therefore, extra-epithelial cells (e.g. fibroblasts) are needed to remodel the ECM prior to crypt formation. Previously, mesenchymal cells have been reported to be the primary source of laminins in the intestine during development (Li et al., 2007; Fields et al., 2019). It seems likely that pericryptal fibroblasts are responsible for supplying the epithelium with laminin due to their close proximity to the crypt epithelium. These cells (Foxl1+, Gli1+, Pdgfra+ and/or CD34+ fibroblasts) also secrete Wnt ligands and R-spondins, which are essential for crypt homeostasis and would therefore express all the factors necessary for the intestinal niche (Degirmenci et al., 2018; Greicius et al., 2018; McCarthy et al., 2020b; Shoshkes-Carmel et al., 2018; Stzepourginski et al., 2017).

We saw a dramatic increase in the expression of basement membrane components in the mesenchyme around birth, supporting earlier observations (Simo et al., 1991; Simon-Assmann et al., 1990). The increase in laminin deposition at birth supports the drastic morphological changes that occur at this stage with crypt formation; thereafter, the expression level decreases as the crypt morphology is maintained through adulthood. This coincides with the expression of *Itga6*, a known laminin receptor, in the intervillus cells and nascent crypts (Lee et al., 1992; Simon-Assmann et al., 1994). We also detected upregulation of other integrins (e.g. *Itga3*) *in vivo* and we speculate that deletion of *Itga6* over longer time periods would be compensated by other integrins. An *Itga6-*knockout mouse has been reported and develops normal crypts; however, it is susceptible to colitis and adenoma formation at later stages (De Arcangelis et al., 2017). Nevertheless, we were able to interfere with the ITGA6 receptor *in vitro* by using a neutralising antibody, highlighting its central role. In recent years, several studies have defined the mechanical properties for stem cell proliferation and cell compartmentalisation (Gjorevski et al., 2016; Engler et al., 2006). Our study showed that changing ECM components while maintaining a similar stiffness affects the epithelium via laminin signalling through the ITGA6 receptor, and points away from a pure physical explanation of the matrix (e.g. changed stiffness) as the responsible factor for the observed effects. Therefore, a localised ECM-epithelial cell signalling is potentially more crucial for stem cell specification and crypt formation. Overall, our study highlights a central role of the ECM in controlling intestinal stem cells and crypt morphol­ogy during development and in the adult.

## MATERIALS AND METHODS

### Mice and crypt isolation

Crypts were isolated from wild type and Lgr5–EGFP–IRES–CreERT2 mice following previously described methods (Barker et al., 2007; Sato et al., 2009). *In vivo* experiments performed in Amsterdam were approved by the Animal Experimentation Committee at the Academic Medical Center in Amsterdam (ALC102556 and Lex 274, WP 17-1884-1-01) and performed according to national guidelines. For Lamc1loxP mouse experiments, husbandry and research protocols were carried out in accordance with protocol AUA00003140, which was approved by The Medical College of Wisconsin's Institutional Animal Care and Use Com­mittee (IACAUC). Ubc-CreERT2 Lamc1loxP/loxP mice were induced with four intraperitoneal injections of 1 mg/ml tamoxifen on four consecutive days (Metzger et al., 1995; Ruzankina et al., 2007; Chen and Strickland, 2003). Mouse tissue was harvested 21 days after first injection.

### Organoid growth in Matrigel

Small intestinal crypts were isolated and cultured as previously described (Sato et al., 2009). Isolated crypts (via EDTA 2 mM) were embedded in growth factor-reduced Matrigel (Corning, 356231). Medium [Advanced DMEM/F12 (ADF), Thermo Fisher Scientific, 12634010] was supplemented with EGF (50 ng/ml, TEBU-BIO, 315-09-A), conditioned medium of Noggin (10%) and conditioned medium of Rspo1 (20%). For the first 48 h after initial purification, the medium was supplemented in addition with the GSK-3 inhibitor CHIR-99021 (5 µM, Axon Medchem BV, Axon 1386) and the Rock inhibitor Y-27632 (10 µM, Sigma-Aldrich, Y0503). The culture medium was changed every other day. Wnt3a-conditioned medium was produced from L-Wnt3a cells in 10% fetal bovine serum and was added at 50% to ENR medium (WENR).

### Cell growth on collagen

Mouse small intestinal cells were grown on collagen as previously described (Wang et al., 2017). First, mouse intestinal organoids grown in Matrigel were released from Matrigel by incubation with Cell Recovery solution (BD Biosciences, 354253) for 30 mins on ice and then mechanically dissociated before being placed on a collagen layer. Collagen rat tail type I (Corning, 354236) layer was prepared and neutralised to a concentration of 1 mg/ml. The collagen mixture was added to a six-well plate (1 ml/well) and left to gelate at 37°C for 1 h. Cells collected from Matrigel cultures were resuspended in medium and plated on top of the hydrogel. For the initial 48 h after passaging, the medium was supple­mented with Y-27632 (10 µM). The medium was changed every other day. Cells were collected 6 days after plating in 1 ml PBS and incubated with 60-80 µl collagenase IV (10 mg/ml, Sigma-Aldrich, C5138-1G) at 37°C for 10 min, then washed three times in 10 ml PBS. Cell pellets were resuspended in culture medium and split into a ratio of one well/two to three wells.

### Cell growth on collagen:basement membrane com­ponents

Higher concentrations of collagen IV (Corning, 354233, 1 mg/ml) were achieved by freeze drying the sample in a lyophiliser (ZIRBUS technology) at 0.05 mbar and −80°C for a period of 72 h. The dried collagen IV was dissolved in PBS at a final concentration of 2 mg/ml. Collagen I and the basement membrane components Matrigel, laminin (Thermo Fisher Scientific, 10256312) or collagen IV were mixed to create the desired concentrations and added to a 12-well plate (0.5 ml/well), with a final collagen I concentration of 1 mg/ml. Cells grown on collagen I (passage number 2-5) were collected and washed following the previously mentioned procedure. The pellet was resuspended in 4 ml of medium and 1 ml was plated on top of each well. For the initial 48 h after passaging, the medium was supplemented with Y-27632 (10 µM). The medium was changed every other day (ENR). Cells were collected 4 days after passaging in 1 ml PBS and incubated with 60 µl collagenase IV (10 mg/ml) for 10 min at 37°C, then placed on ice for 40 min before pelleting. For blocking of ITGA-6, collagen-grown cells were collected and incubated with an ITGA-6 neutralizing antibody (Merck Millipore, MAB1378, clone NKI-GoH3, 40 µg/ml) for 60 min at 37°C before plating on hydrogels. Medium was renewed every 48 h (ENR) with newly added antibodies and RNA was harvested on day 4.

### Immunofluorescence

Immunofluorescence staining was used to reveal the cellular composition of Matrigel, collagen I and collagen:basement membrane cultures. Samples were fixed in 4% formaldehyde for 1 h, permeabilised with 0.2% Triton-X in PBS for 1 h and blocked with Antibody Diluent (ImmunoLogic, VWRKUD09-999) for 30 min. The following antibodies were used: anti-lysozyme EC 3.2.1.17 (1:200, DAKO, A0099), anti-aldolase B EPR3138Y (1:200, Abcam, ab75751) and anti-Ly6a-APC (1:200, eBioscience, 17-5981-81).

Snap-frozen mouse intestinal tissue (E19, P0 and adult) were cut at a thickness of 10 μm using a cryostat and transferred onto Superfrost microscope slides. Sections were fixed with 4% paraformaldehyde for 10 mins at room temperature, permeabilised with 0.2% Triton-X 100 for 10 mins and blocked with Antibody Diluent for 10 min. Slides were incubated with anti-laminin polyclonal antibody (1:200, Thermo Fisher Scientific, PA5-22901) overnight at 4°C. Images were taken using the Leica TCS SP8 X microscope.

### RNAscope

Formalin-fixed paraffin-embedded (FFPE) slides (5 μm) were prepared and stained according to the manufacturer's instruction with RNAscope Detection kit 2.5 (Brown) with the following probes, Mm-Lamc1 (Advanced Cell Diagnostics, 517451), Mm-Col4a1 (Advanced Cell Diagnostics, 412871) and Mm-Itga6 (Advanced Cell Diagnostics, 441701).

### Flow Cytometry and fluorescence-activated cell sorting

Cells grown on collagen were collected in 0.5 ml PBS and incubated with 80 µl of collagenase IV (10 mg/ml) for 10 min at 37°C. After two wash steps with PBS, the cells were dissociated into single cells by incubation with 1 ml TrypLE Express (Invitrogen, 12604-013) for 15 min at 37°C with mechanical dissociation after 10 min. Cells were then incubated with anti-mouse Ly6a-APC (1:1000, eBioscience, 17-5981-81) for 30 min on ice. For sorting and plating of cells, ∼10,000 cells/25 µl Matrigel/well were plated and incubated with CHIR-99021 (5 µm) and Y-27632 (10 µM) for the first 48 h.

### Single-cell RNAseq

Organoids grown in Matrigel and cells grown on collagen, both derived from the same organoid line, were collected 4 days after passaging and digested with TrypLE (Invitrogen) for 20 min at 37°C. Cells were passed through a cell strainer with a pore size of 20 μm. Cells were sorted (3000 single, living cells) by fluorescence-activated cell sorting (FACS/SONY sorter), manually counted and adjusted, and processed using 10× Chromium Single Cell 3′ Reagent Kits v3 library kit (v3.1 Chemistry Dual Index). Reads were de-multiplexed, aligned to the GRCm38/mm10 reference genome (‘refdata-cellranger-mm10-3.0.0’) and counted using the 10× Genomics Cellranger software (v3.1.0). Raw count matrices were analysed with the Seurat library in R (Stuart et al., 2019). Cells expressing less than 1000 genes or more than 15% mitochondrial genes were removed.

### Organoid growth in laminin

Mouse small intestinal organoids were grown in Matrigel as previously described (Sato et al., 2009). Organoids were released from Matrigel by incubation with Cell Recovery solution (BD Biosci­ences, 354253) for 30 min on ice, washed twice in cold PBS and then mechanically dissociated before being embedded in High Concentration Mouse Laminin/Entactin domes (Corning, DLW354259). The medium was changed every other day (ENR).

### RNAseq

Organoids grown in Matrigel were derived from three individual mice (*n*=3) and subsequently grown on collagen supplemented with ENR or WENR, in collagen with WENR and in Matrigel supplemented with ENR or WENR, and were collected for RNA isolation 4 days after passaging. The quality was assessed using the Agilent RNA ScreenTape Assay. RNAseq libraries were prepared with the KAPA mRNA Hyper Prep Kit (KAPA Biosystems) using 550 ng of total RNA and sequenced on an Illumina Hiseq4000 (single read, 50 bp). The reads were aligned to the GRCm38/mm10 reference genome via Rsubread and the reads quantified by featurecounts (Liao et al., 2019, 2014). The raw counts were further analysed with DESeq2 and gene set enrichment analysis was used with the fgsea package (Love et al., 2014; Korotkevich et al., 2021 preprint). The RNAseq dataset of epithelial, fetal intestine (GSE115541, Kumar et al., 2019) was similarly analysed with DESeq2.

### Stiffness determination by Young's Modulus

Young's modulus was determined with a displacement-controlled nanoindenter (Piuma, Optics 11). A spherical probe with a radius of 50 µm and a cantilever stiffness of 0.5 N/m. All measurements were performed with a 1 mm layer of hydrogel covered with PBS according to the manufacturer’s protocol.

### Tissue clearing

Tissue clearing was realised fol­lowing the Clearing enhanced 3D microscopy (Ce3D) protocol (Li et al., 2007). Briefly, 20 ml of Ce3D clearing solution was prepared in a fume hood by dissolving Histodenz [86% (w/v), Sigma-Aldrich] in 40% N-methylacetamide (Sigma-Aldrich, M26305) in PBS. The mixture was placed at 37°C for 72 h or until fully dissolved. Triton-X 100 (0.1% v/v) and 1-thioglycerol (0.5% v/v, Sigma-Aldrich) were added to the dissolved mixture. Mouse intestinal tissue (E19, P0 and adult) was isolated, cut open longitudinally, pinned and fixed overnight in 4% paraformaldehyde at 4°C. Fixed tissues were incubated in a blocking buffer containing 1% bovine serum albumin and 0.3% Triton-X 100 overnight on a shaker. Tissues were then incubated with laminin (1:100, Thermo Fisher Scientific, PA5-22901) and EpCAM (1:100, Biolegend, 324221) in Ce3D solution for 72 h on a shaker. Tissues were washed with PBS and incubated with Ce3D clearing solution for 48 h on a shaker. Cleared tissues were incubated with Hoechst (1:1000) for 8 h before mounting with Ce3D solution and imaging using the Leica TCS SP8 X microscope.

### qRT-PCR

Cells cultured in Matrigel, on collagen or on collagen:basement membrane hydrogels were collected 4 days after passaging following the previously described protocols. Mouse intestine whole tissue, the mesenchymal fraction and the epithelial fraction were isolated and digested following previously reported procedures. RNA was isolated using the NucleoSpin RNA kit (BIOKE, MN740955250) following the manufacturer's guidelines. Reverse transcription was performed using the SuperScript III First-Strand Synthesis Mix (Thermo Fisher Scientific, 18080085) on isolated RNA to obtain complementary DNA (cDNA). Quantitative reverse transcription PCR (qRT-PCR) was performed using the SYBR green detection system (QIAGEN, 218073) and each sample was run with two technical replicates. *Rpl37* was used for normalisation. The primer sequences are provided in Table S1.

### qRT-PCR of Lamc1 mouse model

Intestinal epithelium and mesenchyme were separated by incubation of tissue frag­ments in ice-cold BSS buffer (1 mM KCl, 96 mM NaCl, 27 mM sodium citrate, 8 mM KH_2_PO_4_, 5.6 mM Na_2_HPO_3_, 15 mM EDTA) containing protease and phosphatase inhibitors at 4°C with vigorous shaking for 30 min. Sheets of mesenchyme were then removed with forceps. Total RNA from epithelial cells was treated with EZ-DNase (Invitrogen). Superscript VILO master mix (Invitrogen) was used to synthesise cDNA. TaqMan assays and TaqMan Gene Expression Master Mix (Applied Biosystems) were used to measure expression of the following genes: LamC1 (Mm00711820_m1), Anxa1 (Mm00440225_m1), Col4a1 (Mm01210125_m1), Ly6a (Mm00726565_s1), Msln (Mm00450770_m1), Lyz1 (Mm00657323_m1), Olfm4 (Mm01320260_m1), and LGR5 (Mm00438890_m1). GAPDH (4351309) was used for normalisation.

### Statistical analysis

Standard statistical analysis was calculated using Graphpad Prism 8. Sample sizes, statistical tests and definitions of error bars are indicated in the figure legends. Statistical significance of two group comparisons was determined by two-tailed unpaired Student's *t*-test and that of three or more groups was determined by a simple linear regression model.

## Supplementary Material

10.1242/biolopen.059544_sup1 Supplementary information
