# Supplementary material for: The extracellular matrix controls stem cell specification and crypt morphology in the developing and adult mouse gut
Source: Biol Open. 2022 Nov 23;11(12):bio059544. doi: 10.1242/bio.059544 (PMC9713296; doi:10.1242/bio.059544)
Supplement: Supplementary information [file biolopen-11-059544-s1.pdf]

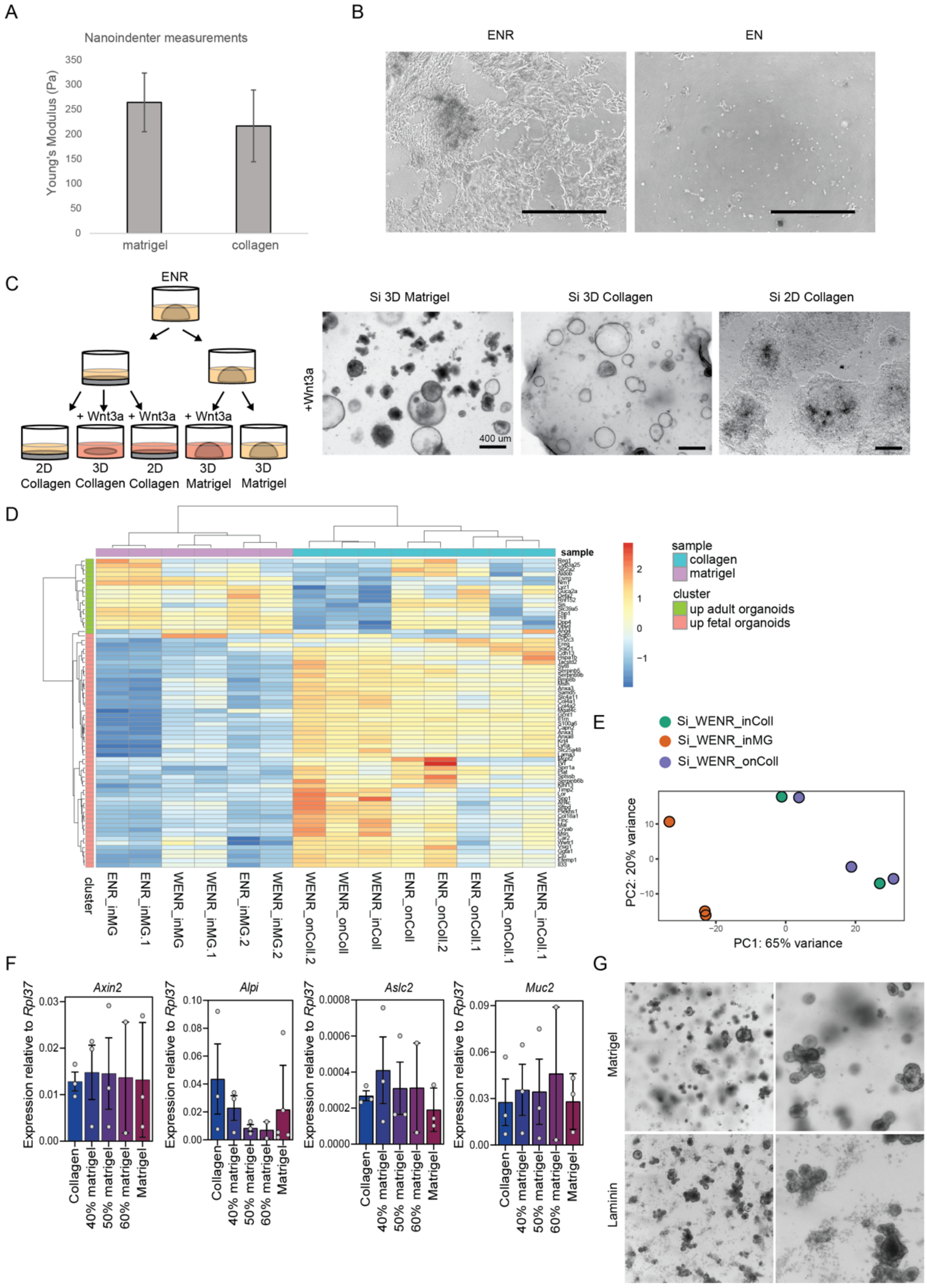

Fig. S1. A, Stiffness measurement (Young's modulus) was measured with a Nanoindenter (Piuma - Optics 11) on collagen or Matrigel hydrogel in PBS at room temperature. Technical measurements from the same sample were averaged, and repeated with new hydrogels (n=2). **B**, collagen cultures require the addition of R-spondin for long-term growth. Images after 1 passage with EN (EGF, Noggin) or ENR (EN+R-spondin). Scale bar = 1000um. **C**, Schematic of experimental design of RNAseq of 2D and 3D collagen cultures, with/without addition of Wnt3a-CM (50%). Scale bar = 400um. **D**, Heatmap of Mustata fetal gene signature (13), selected for highly variable genes (sd >1.5), shows that all collagen cultures are enriched for fetal-like genes, whereas Matrigel cultures are enriched for adult-genes. Addition of Wnt3a decreases expression of adult-genes in collagen cultures. ENR = EGF, Noggin, R-spondin. WENR = Wnt3a-CM (50%) + ENR. **E**, PCA of Matrigel culture, 2D and 3D collagen culture, all with addition of Wnt3a shows that collagen cultures cluster together, separated from the Matrigel culture, n = 2-3 samples per group, derived from different mice. **F**, Expression of Wnt target gene (Axin2), enterocyte gene (Alpi), stem cell marker (Ascl2) and goblet cell marker (Muc2). Each dot represents an independent sample, bar height = mean, error bar = s.e.m. **G**, Organoid forming capacity of intestinal cells in both Matrigel and pure laminin domes.

Biology Open • Supplementary information

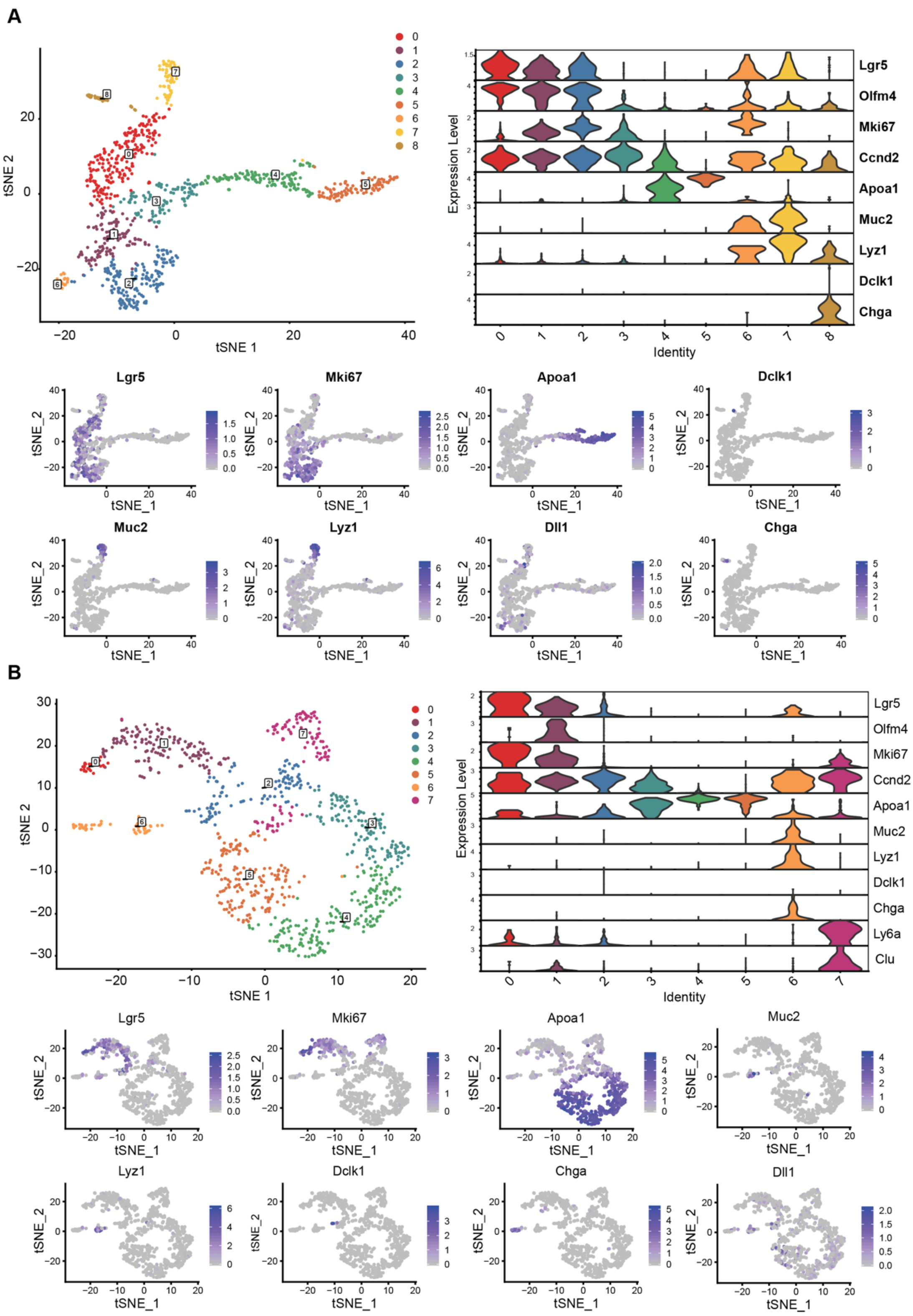

Fig. S2. A, scRNAseq of Matrigel cultures. Clusters as described and as shown in Fig.2a. Violinplot shows expression of cell lineage genes per cluster. Expression of individual cell lineage genes overlaid in tSNE-plot. **B**, scRNAseq of collagen cultures. Clusters as described and as shown in Fig. 2b. Violinplot shows expression of cell lineage genes per cluster. Expression of individual cell lineage genes overlaid in tSNE-plot.

Biology Open • Supplementary information

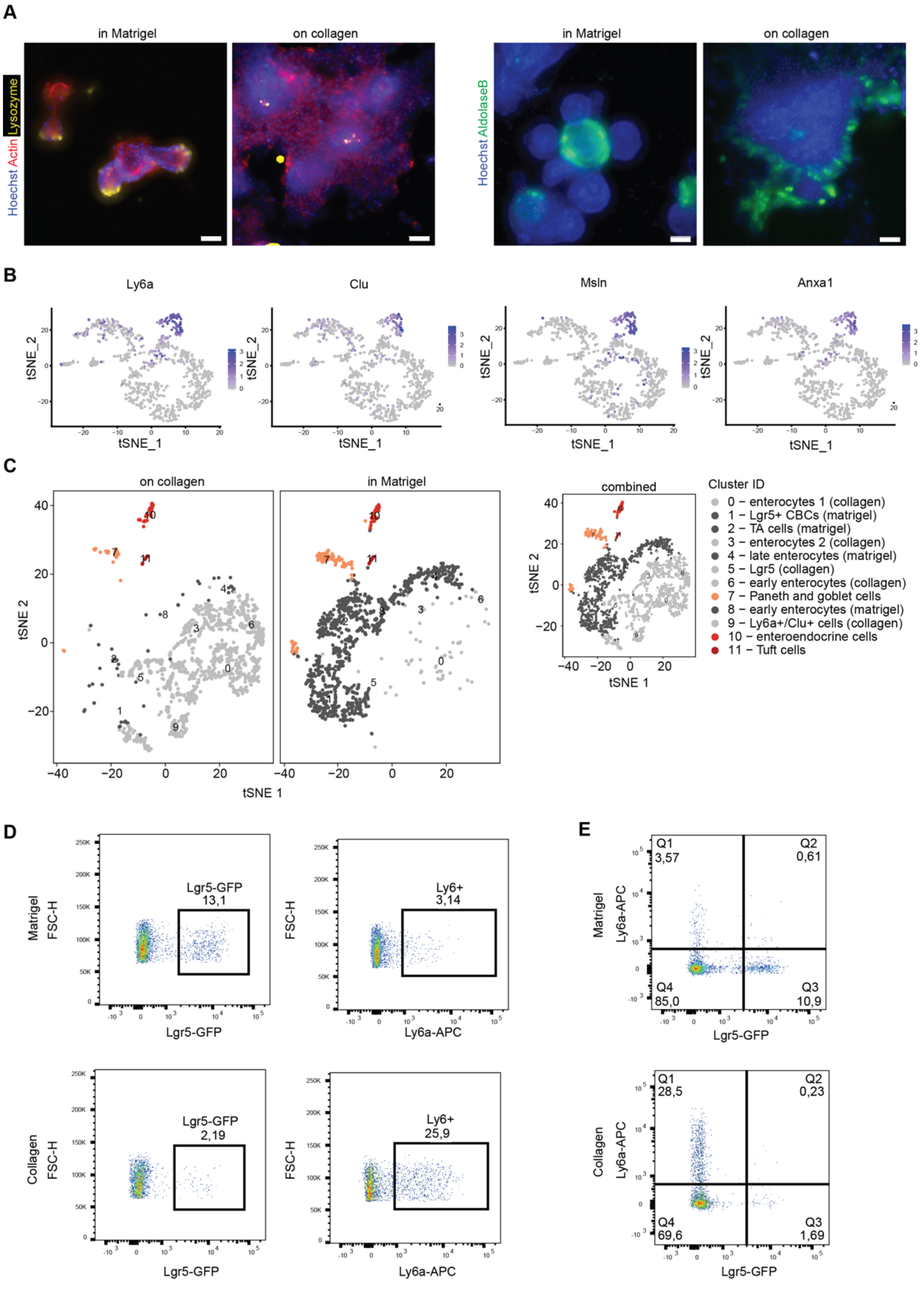

Fig. S3. A, Differentiated Paneth cells (Lysozyme+) and enterocytes (Aldolase B+) are present in Matrigel as well as in collagen cultures. Scale bar = 100um. **B**, Cells expressing fetal-like genes (Ly6a, Clu, Msln, Anxa1) are highly enriched in cluster 7. **C**, Clustering analysis of cells from Matrigel and collagen combined (small insert). For visualisation, tSNE plot is split by origin of cells, either Matrigel or collagen. Dark grey points are nearly exclusively derived from Matrigel, and light grey dots are nearly exclusively derived from collagen. Secretory cells (orange-red) cluster together indicating a similar transcriptional profile irrespective of the matrix they are grown in. **D**, Representative flow cytometry for Lgr5-GFP and LY6A-APC on small intestinal cultures from Matrigel and collagen (n = 2). **E**, There were no cells stained positive for LY6A that also expressed Lgr5-GFP (Q2) indicating independent cell lineages.

Biology Open • Supplementary information

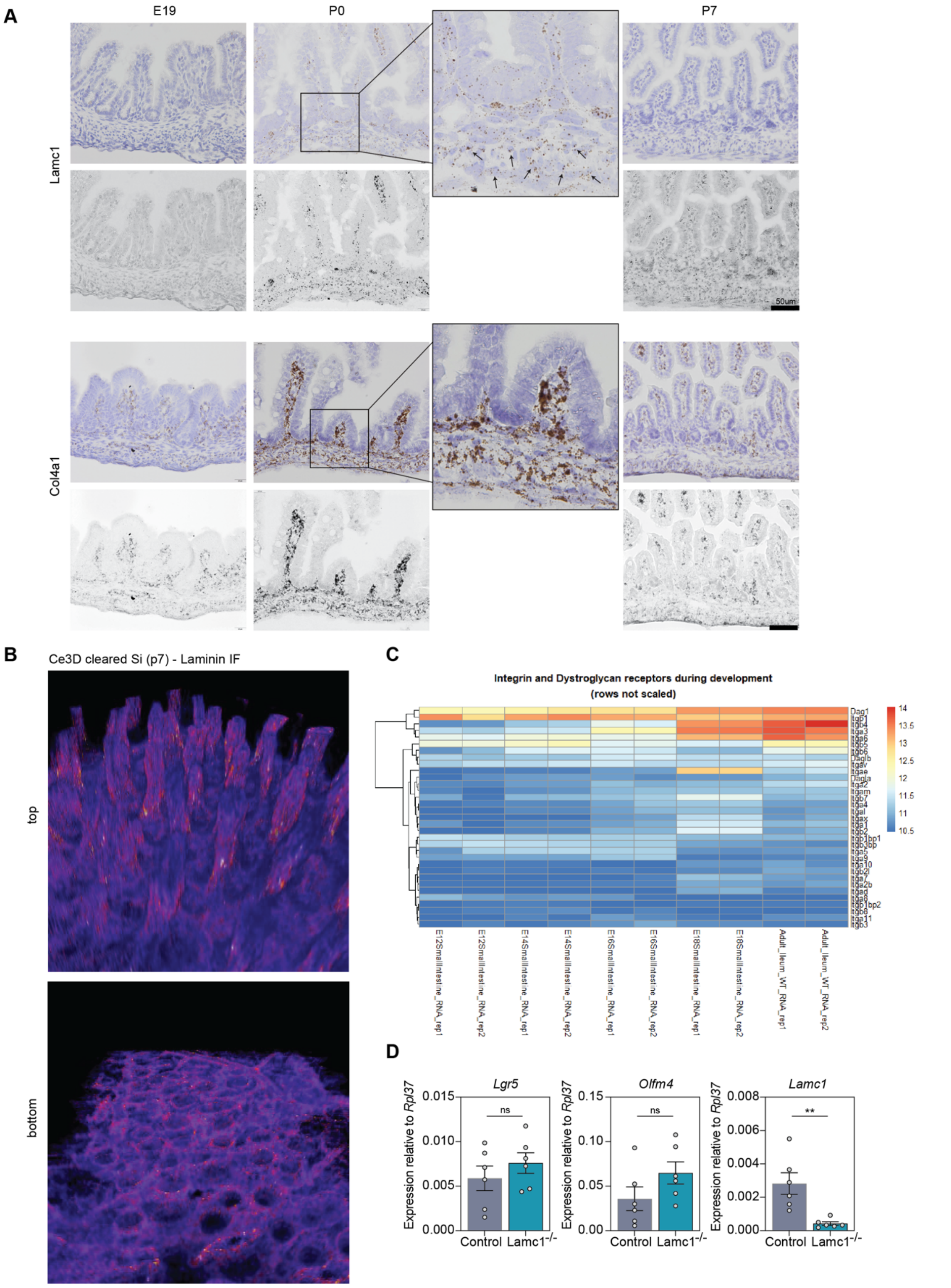

Fig. S4. A, RNA in situ (RNAscope). RNA in situ hybridization for Laminin C1 and Col4a1 shows highest expression at birth (p0 -as shown in Fig. 3h) and is nearly exclusively found in the mesenchyme. Scale bar = 50um. Black arrows indicate Lamc1 signal in mesenchyme. **B**, Cleared tissue (with Ce3D protocol) from small intestine 7 days after birth (P7) shows underlying laminin network, in the villi (top) and surrounding nascent crypts (bottom). **C**, RNAseq analysis of small intestinal epithelial cells during development (GSE115541) shows expression of genes encoding Integrins and Dystroglycans. Top cluster of genes shows change in expression between E12-E16 and E18-Adult and is highlighted in Figure 4A. **D,** Expression of stem cell markers following in vitro knockout of *Lamc1* in murine organoids. Unpaired t test, ** P<0.005, ns non-significant.

Biology Open • Supplementary information

Table S1. qRT-PCR primers

Gene	Forward primer	Reverse primer
Col4a1	CAGGACTTGGGTACGGCTG	CTTCACAAACCGCACACCTG
Col4a2	GTGTCGCAGGGAGTCCGAA	GCTCGATCCCTTCCTAACCTCA
Basp1	GCACCCAAAGCCGAACTCC	GTCGTTCACATTGTAGCCCTTC
Anxa1	GGAGAAAGGGGACAGACGTG	TGGCACACTTCACGATGGTT
Ly6a	AGGAGGCAGCAGTTATTGTGG	CGTTGACCTTAGTACCCAGGA
Msln	GGTCCTGTGGAAGTCCCATC	GGGGAGACTGGCAAAGTCAG
Lyz1	GAGACCGAAGCACCGACTATG	CGGTTTTGACATTGTGTTCGC
Lyz2	ATGGAATGGCTGGCTACTATGG	ACCAGTATCGGCTATTGATCTGA
Lgr5	TTCGTAGGCAACCCTTCTCT	TCCTGTCAAGTGAGGAAATTCA
Olfm4	GCCACTTTCCAATTTCAC	GAGCCTCTTCTCATACAC
Axin2	CCATGACGGACAGTAGCGTA	CTGCGATGCATCTCTCTCTG
Alpi	CACAGCTTACCTGGCACTGA	GGTCTCTGACGACAGGGGTA
Ascl2	CTACTCGTCGGAGGAAAG	ACTAGACAGCATGGGTAAG
Muc2	CTGACCAAGAGCGAACACAA	CATGACTGGAAGCAACTGGA
Lama1	CAGCGCCAATGCTACCTGT	GGATTCGTACTGTTACCGTCAC A
Lama2	TCCCAAGCGCATCAACAGAG	CAGTACATCTCGGGTCCTTTTTC
Lama4	AGTGTCGCAAGCAAGATCCA	TGCTTCCGAGGTAGAGGACA
Lama5	GCTGGCGGAGATCCCAATC	GTGTGACGTTGACCTCATTGT
Lamb1	GAAAGGAAGACCCGAAGAAAAGA	CCATAGGGCTAGGACACCAAA
Lamb2	CCCCGTCCTTGGATGTACCT	CAGTAGGGTTGAGGGCTATGC
Lamc1	GCCGCCAATGTGTCAATCAC	GCCTCAGCTGAAGTCTCGTT
Intga6	TGCAGAGGGCGAACAGAAC	GCACACGTCACCACTTTGC
Rpl37	GTAAGCGTCGCAACAAGACG	TTTAGGTGCCTCATCCGACC

Biology Open • Supplementary information
